# Sustained antimicrobial activity and reduced toxicity of oxidative biocides through biodegradable microparticles

**DOI:** 10.1016/j.actbio.2017.10.001

**Published:** 2017-12

**Authors:** Panagiotis Sofokleous, Shanom Ali, Peter Wilson, Asma Buanz, Simon Gaisford, Dharmit Mistry, Adrian Fellows, Richard M. Day

**Affiliations:** a Division of Medicine, University College London, Gower Street, London WC1E 6BT, UK; b Environmental Research Laboratory, University College Hospital, 235 Euston Road, London NW1 2BU, UK; c School of Pharmacy, University College London, Brunswick Square, London WC1N 1AX, UK; d GAMA Healthcare Ltd, 2 Regal Way, Watford WD24 4YJ, UK

**Keywords:** Thermally induced phase separation, Oxidative biocide, Antimicrobial agent, Resistance, Antibiotics

## Abstract

The spread of antibiotic-resistant pathogens requires new treatments. Small molecule precursor compounds that produce oxidative biocides with well-established antimicrobial properties could provide a range of new therapeutic products to combat resistant infections. The aim of this study was to investigate a novel biomaterials-based approach for the manufacture, targeted delivery and controlled release of a peroxygen donor (sodium percarbonate) combined with an acetyl donor (tetraacetylethylenediamine) to deliver local antimicrobial activity via a dynamic equilibrium mixture of hydrogen peroxide and peracetic acid. Entrapment of the pre-cursor compounds into hierarchically structured degradable microparticles was achieved using an innovative dry manufacturing process involving thermally induced phase separation (TIPS) that circumvented compound decomposition associated with conventional microparticle manufacture. The microparticles provided controlled release of hydrogen peroxide and peracetic acid that led to rapid and sustained killing of multiple drug-resistant organisms (methicillin-resistant Staphylococcus aureus and carbapenem-resistant Escherichia coli) without associated cytotoxicity *in vitro* nor intracutaneous reactivity *in vivo*. The results from this study demonstrate for the first time that microparticles loaded with acetyl and peroxygen donors retain their antimicrobial activity whilst eliciting no host toxicity. In doing so, it overcomes the detrimental effects that have prevented oxidative biocides from being used as alternatives to conventional antibiotics.

**Statement of Significance:**

The manuscript explores a novel approach to utilize the antimicrobial activity of oxidative species for sustained killing of multiple drug-resistant organisms without causing collateral tissue damage. The results demonstrate, for the first time, the ability to load pre-cursor compounds into porous polymeric structures that results in their release and conversion into oxidative species in a controlled manner. Until now, the use of oxidative species has not been considered as a candidate therapeutic replacement for conventional antibiotics due to difficulties associated with handling during manufacture and controlling sustained release without causing undesirable tissue damage. The ultimate impact of the research could be the creation of new materials-based anti-infective chemotherapeutic agents that have minimal potential for giving rise to antimicrobial resistance.

## Graphical abstract

**Figure:**
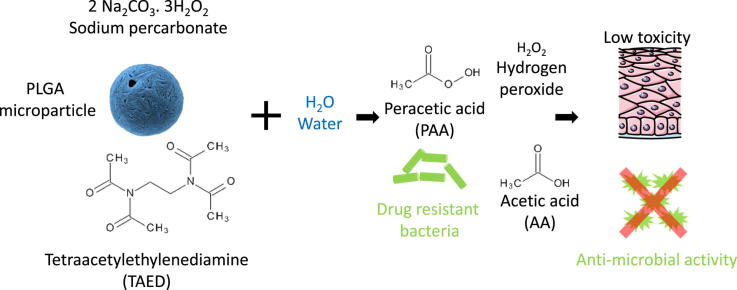

## 1 Introduction

Many antibiotics, antiseptics and disinfectants developed to date are susceptible to bacterial resistance [1], [2], [3]. There is, therefore, an urgent need to find alternative antimicrobial compounds that provide broad spectrum activity to which resistance is less likely to be present. Re-evaluation of existing high energy oxidative species, such as hydrogen peroxide (H_2_O_2_) and peracetic acid (PAA), with well-established biocidal properties could provide a range of new therapeutic products [2]. PAA does not give rise to bacterial resistance, whereas some resistance can occur to H_2_O_2_ for organisms producing catalase and dismutase enzymes [4], [5]. However, when used alone, these low-molecular weight oxidising agents offer limited clinical therapeutic potential since they pass easily through cell membranes and at high local concentrations lead to cell death, causing unwanted off-target tissue damage [6], [7]. To date, safe and efficacious delivery of oxidative species into sites of infection without causing unacceptable tissue damage has proven to be challenging [8], [9], [10].

**Fig. 1:**
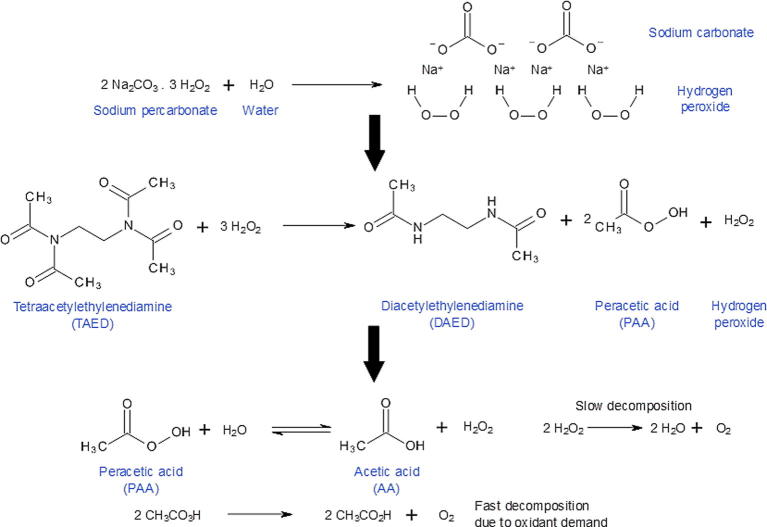
Decomposition of TAED and SP compounds into secondary products. SP decomposes into H_2_O_2_ that reacts with TAED through hydrolysis and perhydrolysis to produce triacetylethylenediamine (TriAED) and diacetylethylenediamine (DAED), resulting in the release of two moles of peracetic acid (PAA) or acetic acid (AA).

Controlled delivery of potentially toxic compounds for use in other areas of biomedicine (e.g. cancer chemotherapy) has seen development of a range of innovative particulate-based delivery systems that provide localised, sustained release of compounds at sub-toxic levels [11], [12], [13]. However, loading precursors of oxidative species, such as tetraacetylethylenediamine (TAED) and sodium percarbonate (SP), into delivery systems is particularly challenging because exposure to aqueous environments typically associated with conventional microparticle manufacture processes results in rapid decomposition into secondary products (Fig. 1).

Chemical degradation of precursor compounds during manufacture may also be affected by pH, ionic strength, light and environmental temperature [14], [15]. To achieve effective localised and sustained release, physicochemical properties of the delivery system must be compatible with the bioavailability/bioactivity of the entrapped pre-cursor compound and capable of releasing the oxidative species in a controlled manner without causing toxicity to surrounding tissue [13], [16], [17].

SP and TAED are non-toxic solid compounds, if used at low concentrations, with exceptional storage stability. SP forms sodium carbonate (SC) and H_2_O_2_ when it comes into contact with aqueous media [18]. H_2_O_2_ react with TAED through hydrolysis and perhydrolysis to produce triacetylethylenediamine (TriAED) and diacetylethylenediamine (DAED), resulting in the release of two moles of PAA or acetic acid (AA) (see Fig. 1) [19]. As a biocidal system this mixture is more effective than H_2_O_2_ alone in terms of contact time, antimicrobial activity spectrum and activity in environmental conditions where there is a heavy organic load that might attenuate the activity of the oxidative biocide [20], [21].

Synthetic polymer-based devices are widely used in clinic for sustained drug delivery (e.g. Ozurdex, Zoladex, Risperdal Consta). The active ingredient is entrapped in a polymer matrix, such as poly(lactic-co-glycolic acid) (PLGA) [12], [22]. When implanted into the body the active ingredient is gradually released as the polymer degrades [16], [23]. This approach could lend itself to the sustained release of TAED and SP if the compounds can be entrapped without exposure to an aqueous environment during the manufacturing process. Thermally induced phase separation (TIPS) microparticle manufacturing uses lyophilisation to remove residual solvent instead of aqueous-based washing used with conventional solvent-evaporation microparticle manufacturing techniques.

The aim of this was study was to investigate whether entrapping oxidative species precursor compounds into biodegradable polymer microparticles using TIPS technology [24] provides a method for reducing local toxicity of the compounds whilst retaining their antimicrobial activity.

## 2 Experimental section

### 2.1 Preparation of solutions

Poly(lactic-co-glycolic) acid (PLGA) PURASORB 7507 (75:25) polymer (Corbion, Amsterdam, Netherlands) was dissolved in dimethyl carbonate – DMC (Sigma Aldrich, Dorset, UK) overnight to produce a 4% (w/v) polymeric solution. TAED (Warwick Chemicals, Holywell, UK) was mixed with acetonitrile – ACN (Sigma Aldrich) at a concentration of 200 mg/mL and stirred for 1 h using a magnetic stirrer. The TAED + ACN suspension was added to the PLGA solution (11% v/v) and stirred for a further 30 min before use. SP (Solvay, Warrington, UK) was mixed with analytical grade water – AGW (200 mg/mL) and stirred for 1 h using a magnetic stirrer. The SP + AGW suspension was added to the PLGA + DMC solution (11% v/v) and stirred for a further 30 min.

### 2.2 Preparation of TIPS microparticles

TIPS microparticles were prepared using a VAR-D modular microencapsulation unit (NISCO Engineering AG, Zurich, Switzerland; Frequency: 2.75 kHz, Amplitude: 70%) fitted with a sapphire tipped nozzle (150 μm orifice). The flow rate of polymer solution delivered into the unit was 3 mL/min. The polymer droplets were collected in liquid nitrogen (LN_2_) and the solvent removed by lyophilisation for 48 h. The dried microparticles were sieved to a size range of 250–425 μm before being stored under vacuum in sealed glass containers at 4 °C.

### 2.3 Characterization of TIPS microparticles

#### 2.3.1 Scanning Electron Microscopy (SEM)

Microparticles were vacuum-coated with gold/palladium for 120 s and images using JEOL USA JSM-7610F SEM. Size distribution: Morphologi G3 (Malvern Company, Worcestershire, UK) was used to characterize the size and shape of the microparticles using static image analysis.

#### 2.3.2 Differential scanning calorimetry (DSC)

Measurements were performed on a TA Instruments Q2000 DSC (TA Instruments, Waters, LLC, USA) equipped with a refrigerating cooling accessory and using nitrogen as a purge gas at a flow rate of 50 mL/min. Cell constant and enthalpy calibration was performed with standard indium according to the manufacturer’s instructions. Samples were heated in Tzero® aluminium pans and lids at 10 °C/min–100 or 200 °C. Data were collected using TA Advantage software and analysed using TA Universal Analysis 2000 (TA Instruments, Waters, LLC, USA). Transition temperatures were reported as extrapolated onset and/or peak point calculated along with the enthalpy using the sigmoidal peak integration function. Glass Transition (Tg) were reported as the midpoint at the inflection.

#### 2.3.3 Thermogravimetric analysis (TGA)

TA Instruments Discovery TGA (TA Instruments, Waters, LLC, USA) was used to measure weight change upon heating at 10 °C/min up to 350 °C using nitrogen as a purge gas at a flow rate of 25 mL/min. The machine was calibrated for weight and temperature according to the manufacturer’s instructions. Data collection and analysis were performed using TA Instruments Trios software (TA Instruments, Waters, LLC, USA).

#### 2.3.4 Powder X-ray diffraction (PXRD)

Measurements were performed on Rigaku Mini 600 (Rigaku, Tokyo, Japan), operated with Cu Kα radiation (1.5418 Å) at 40 kV and 15 mA), between 5 and 35° at 2°/min speed and 0.02° step size.

### 2.4 Loading efficiency for TAED and SP in TIPS microparticles

#### 2.4.1 TAED

The method published by Wissenschaftliche Verlagsgesellschaft mbH, Stuttgart, Germany was followed to detect the entrapment efficiency of TAED in the TIPS microparticles [25]. A standard calibration of 0–100 ppm was prepared in acetonitrile. Approximately 10 mg of the TIPS microparticles were dissolved in 100 mL of acetonitrile. The standards and samples were run on a gas chromatography–mass spectrometer (GC-MS) with an HP5-MS UI 30 m × 0.250 mm × 0.25 μm [19091S-433UI] column.

#### 2.4.2 SP

The entrapment efficiency of SP in the microparticles was measured using a spectrophotometric method developed in-house. A calibration curve was prepared using SP (Acros Organics and previously titrated as 74.0% w/w active SP) dissolved in ultra-pure water. Specific volumes of the SP solution containing known quantities of SP were added to ammonium molybdate solution, acetate buffer and potassium iodide solution, which were then incubated in the dark for 10 min and the optical density measured at 352 nm after subtracting the blank background. A known quantity of the microparticles loaded with SP were washed with acetonitrile. The sample was centrifuged, the acetonitrile removed and a fresh volume added. The sample was washed a total of three times. The remaining SP residue was added to ammonium molybdate solution, acetate buffer and potassium iodide solution, which were then incubated in the dark for 10 min and the optical density measured at 352 nm after subtracting the background from the blank.

### 2.5 Microbiology studies

#### 2.5.1 Preparation of test organisms

*Target organisms:* Clinical isolates of carbapenem-resistant *E. coli* (CREC) and methicillin-resistant Staphylococcus aureus (MRSA; EMRSA-15 variant B1). *Preparation of bacteria:* 10 µL of test bacteria was transferred with a loop to sterile nutrient broth (10 mL; Oxoid, UK), mixed thoroughly and incubated aerobically at 37 °C for ∼24 h (±4 h). Broth cultures were centrifuged at 3000 rpm (1500×*g*; Jouan CR3i centrifuge: Thermo, UK) for 10 min and the remaining pellet re-suspended in 10 mL of sterile distilled water (DW). During time-kill assays, the PAA antimicrobial agent was inactivated after the appropriate contact time by the addition of a neutralising solution [3% (w/v) Tween 80, 0.3% (w/v) Lecithin, 1.0% (w/v) Sodium thiosulfate, 1.5% (w/v) K_2_HPO_4_, KH_2_PO_4_ 0.05% (w/v), 1% (w/v) Poly-[sodium-4-styrenesulfonate], 0.1% (v/v) Triton® X100 (Sigma-Aldrich, UK) and prepared in phosphate-buffered saline (PBS) solution (Oxoid, UK)]. Neutralising solutions were sterilised by autoclaving (121 °C for 15 min) and refrigerated (2–8 °C) until required. All microbiology studies were conducted by the Environmental Research Laboratory, University College Hospital.

#### 2.5.2 Validation of neutraliser efficacy and toxicity

The ability of the neutraliser to quench the antimicrobial activity of microparticles loaded with TAED was confirmed and any toxicity against the test organisms assessed as previously described [26]. An aliquot (80 mg) of the test (TAED + SP) and control (unloaded) microparticle preparation was inoculated with 8 mL sterile DW, mixed at high speed for 30 s and hydrated at room temperature for 1.5 h to allow the antimicrobial to leach into the solution. One millilitre aliquots from the resulting microparticle-leachate (free-of microparticles) was transferred to a separate container. A sterile test tube was dosed with: (A) 1 mL neutraliser + 1 mL microparticle leachate, (B) 1 mL neutraliser A + 1 mL sterile distilled water, 1 mL microparticle leachate (no-antimicrobial) or (D) 2 mL distilled water (control) and incubated at room temperature for 1 min to allow neutralisation of the microparticle-leachate solution. This was inoculated with 1 mL (10^3^ colony-forming units [cfu]) of CREC or MRSA prepared in sterile distilled water. The resulting suspensions were incubated a further 10 min at room temperature before spread-plating 0.1 mL onto Columbia blood agar plates (a simple non-selective medium). Plates were incubated aerobically (CREC, MRSA) at 37 °C for 48 h.

#### 2.5.3 Determination of bactericidal/bacteriostatic nature of TIPS microparticles loaded with SP and TAED

Preparations of control (80 mg: unloaded) and test (40 mg TAED + 40 mg SP – loaded) microparticles were activated by adding 8 mL Eagle’s Minimum Essential Medium (EMEM) (Sigma Aldrich, UK), 10% v/v fetal bovine serum (FBS) (Sigma Aldrich), 2 mM l-Glutamine (Sigma Aldrich) (complete EMEM), mixing by vortex for 30 s and incubating for 1.5 h at room temperature. One mL aliquots of the hydrated microparticle suspension were transferred to individual universal tubes. Each microparticle aliquot was inoculated with 1.0 mL of the test suspension (∼10^6^ cfu bacteria) prepared in sterile phosphate-buffered saline [PBS]) and the suspension mixed by vortex shaking for 30 s. Inoculated suspensions were incubated aerobically and without agitation at 37 °C for a further 6 h after which the antimicrobial activity was stopped by adding 0.1 mL of neutralising solution followed by incubation at room temperature for a further 10 min. After neutralisation, suspensions were centrifuged at 3000 rpm (∼1100×*g*) for 10 min. The resulting supernatant was discarded and the pellet resuspended in 2 mL sterile PBS and washed by vortexing at high speed for 30 s and repeating the centrifugation and wash stages for a total of three washes. The final (washed) pellet was resuspended in 2 mL sterile PBS as previous. One-hundred millilitre aliquots of serial dilutions (1/1, 1/10, 1/100 and 1/1000) of the washed suspensions were spread-plated onto Columbia Blood agar and incubated aerobically at 37 °C for 48 h prior to counting the bacterial cfu formed in the agar plates. Tests were repeated six times.

#### 2.5.4 Minimum inhibitory concentration (MIC) and minimum bactericidal concentration (MBC) of the antimicrobial compounds

The release of oxidative biocides from the TAED precursor loaded microparticles is dependent on the presence of H_2_O_2_ released from the SP-loaded microparticles. Therefore TAED microparticles alone did not have any bactericidal action. Based on the intended combined use of TAED and SP loaded microparticles, the MIC and MBC were performed only for PLGA-TAED-SP microparticles. Preparations of loaded and unloaded microparticles were hydrated with EMEM as described above. Serial dilutions of each suspension (unloaded and TAED + SP microparticles) were prepared in 10% increments ranging from 0% to 100% of the original suspension using sterile nutrient broth (Oxoid, UK). Aliquots (1 mL) of each concentration was transferred to a sterile 24 well microplate (Corning® CellBIND®, Sigma-Aldrich, UK). Overnight cultures of CREC and MRSA were prepared in nutrient broth as previously described after which serial 1/10 dilutions of the initial suspension performed to achieve a stock inoculum concentration of approximately 10^6^ cfu/mL. One millilitre aliquots of the test bacterial inoculum was transferred to each microplate-well pre-dosed with the test antimicrobial suspension and mixed gently by forward/backward pipetting. The microplates were incubated aerobically for ∼24 h at 37 °C after which 0.1 mL of the neutraliser solution was added to each well and incubated at room temperature for 10 min. The quantity of bacteria present was determined by plating 0.1 mL and 0.5 mL from each well onto a blood agar plate and incubated a further 24 h at 37 °C prior to counting the bacteria CFU formed in the agar plates.

#### 2.5.5 Antimicrobial activity of loaded TIPS microparticles

The bactericidal activity of loaded and unloaded microparticles was evaluated using a time-kill suspension-assay based upon BS EN 1276 protocols for antimicrobial efficacy testing of bactericidal agents against CREC and MRSA. Briefly, the microparticles (control: 80 mg unloaded and test: 40 mg TAED + 40 mg SP – loaded) were activated by adding 8 mL complete EMEM, mixed by vortex for 30 s and incubated for 1.5 h at 37 °C. Immediately after hydration, 1 mL of the test bacterial suspension (∼10^6^ cfu CREC or MRSA, prepared in sterile complete EMEM medium was added to 1 mL of complete EMEM medium (this functioned as the interfering substance/soil simulating an organic challenge). The resulting suspension was transferred to 8 mL of the hydrated microparticle suspension and mixed by vortexing for 30 s. Solutions were incubated at 37 °C for contact times of: 0, 1, 2, 4, 6 and 18 h after which bactericidal activity was stopped by transferring 1 mL of the suspension to 1 mL neutralising solution followed by incubation at room temperature for 10 min. Serial dilutions (1/1, 1/10 and 1/100) of suspensions were performed prior to spread-plating 0.1 mL and 0.5 mL aliquots onto Columbia blood agar (CBA) plates. Plates were incubated aerobically at 37 °C for 48 h prior to counting the bacteria CFU formed in the agar plates. Tests were repeated nine times.

To investigate the ability of the loaded microparticles to exhibit longer term antimicrobial activity against CREC and MRSA the incubation period of the microparticles (control: 80 mg unloaded and test: 40 mg TAED + 40 mg SP – loaded) were activated by adding 8 mL complete EMEM, mixed by vortex for 30 s and incubated for 14 days at 37 °C with agitation by vortex for 30 s daily. At pre-determined intervals (i.e. 1, 2, 3, 7 and 14 days) 0.5 mL aliquots of the microparticle suspension was transferred to individual 30 mL containers. Each aliquot was inoculated with 0.5 mL of a bacterial inoculum preparation – (∼10^6^ cfu CREC or MRSA*,* prepared in sterile EMEM) and the suspension mixed by vortex. The microparticle-inoculum mix was incubated at 37 °C for contact times of: 0, 1, 2, 4, 6 and 18 h after which bactericidal activity was stopped by the addition of 0.5 mL neutralising solution followed by incubation at room temperature for 10 min. Serial dilutions (1/1, 1/10 and 1/100) of the neutralised suspensions were spread-plated onto Columbia blood agar plates. Plates were incubated aerobically at 37 °C for 48 h prior to counting the bacteria CFU formed in the agar plates. Tests were repeated six times.

### 2.6 *In vitro* cytotoxicity

Cytotoxicity of microparticles loaded with TAED and SP and the compounds in their raw format were evaluated *in vitro* using the CellTox™ Green cytotoxicity assay (Promega UK) and L929 fibroblasts. The cells were mixed with the CellTox dye according to the kit manufacturer’s instructions, transferred into a black walled 96 well plate (5000 cells per well) and incubated for 24 h a 37 °C, 5% CO_2_. 50 μl of the supernatant was collected from microparticles that had been hydrated sterile complete EMEM medium (10 mg/mL) for pre-determined amounts of time (1.5 h, 1 d, 3 d, 7 d and 14 days) and added to the cells in the 96 well plate. Fluorescence readings were collected from the wells after different periods of incubation (30 min, 90 min, 3 h, 5 h and 24 h; TECAN GENios Multifunction Fluorescence Microplate Reader; excitation wavelength of 485–500 nm and emission of 520–530 nm). Readings from test samples were normalised with lysed samples representing 100% toxicity.

### 2.7 *In vivo* biocompatibility

18 Sprague Dawley male rats (weight 385 ± 15 g) were used to investigate the intracutaneous reactivity of the microparticles. Following anaesthetisation with inhaled isoflurane and administration of an analgesic (Carprofen – 0.1 mL/kg) the fur on the dorsum was trimmed with clippers and then shaved with a disposable razor. The skin was cleaned with chlorhexidine in alcohol before surgery and allowed to dry. Immediately before delivery, the microparticles were mixed into a clear viscous inert hydrogel (50 mg/mL; GranuGEL® ConvaTec, UK) to facilitate delivery through a 16G needle. The rats (n = 6) were randomly assigned to 3 groups to receive a subcutaneous injection of microparticles loaded with either TAED (100 μl), SP (100 μl) or a combination of TAED and SP microparticles (100 μl each; total volume 200 μl). Each animal received a second subcutaneous injections in the dorsum containing an equivalent volume of unloaded TIPS microparticles. The injection site was identified with a permanent marker and photographed. At 7, 14 and 21 days post-delivery, 2 rats from each group were terminated using an intraperitoneal injection of sodium pentobarbitone. The microparticle injection area was trimmed and shaved and the area photographed. The implant site and surrounding 5 mm of tissue was excised and placed into 4% neutral buffered formaldehyde for 24 h fixation. The *in vivo* study was conducted by Northwick Park Institute for Medical Research (PPL 80/2639). The experimental protocol was approved by the Institutional Animal Care and Use Committee of NPMIR.

### 2.8 Histology

The fixed tissue samples were washed in PBS and dehydrated in a graded series of alcohol before being embedded in Paraplast X-tra® (Sigma). Tissue sections (5 µm) were cut from the wax blocks before staining with haematoxylin and eosin. Images were acquired using a Hamamatsu Nanozoomer-HT-2.0 Digital pathology System (NDP).

### 2.9 Statistical analysis

Data were tested for Gaussian distribution and analysed statistically using OriginPro and GraphPad Prism software. For data sets with a Gaussian distribution statistical evaluation was performed by Two-Way ANOVA with Dunnett’s test for multiple comparisons. For data sets with a non-Gaussian distribution statistical evaluation was performed by the Friedman test.

## 3 Results

### 3.1 Characterization of TIPS microparticles

**Fig. 2:**
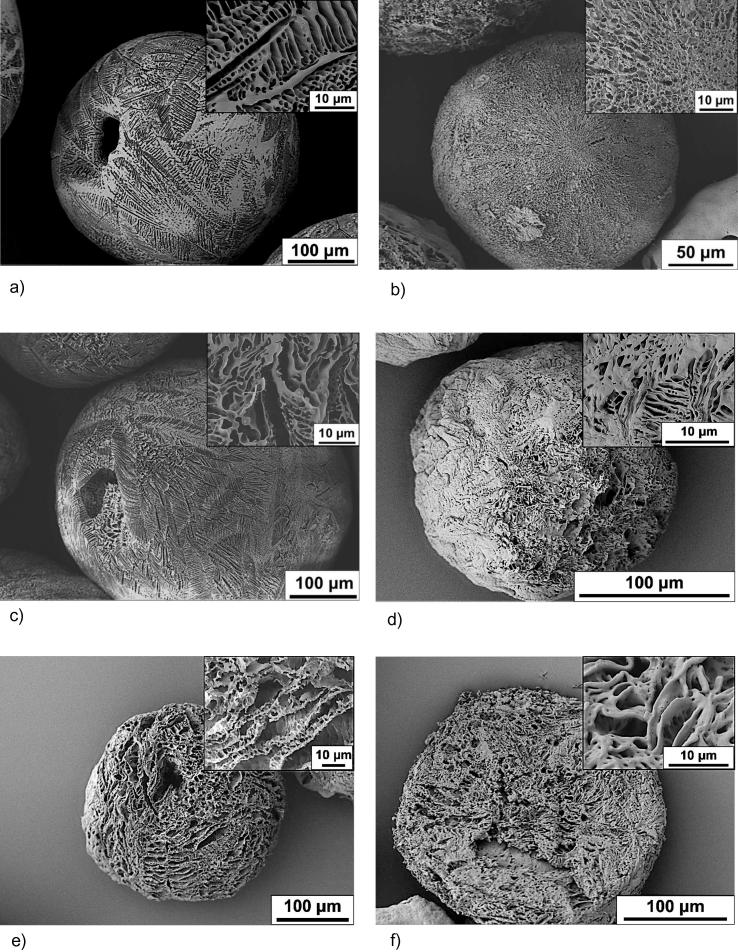
Scanning electron microscopy (SEM) images showing TIPS microparticles before and after *in vitro* hydration for 28 days. (a) Unloaded, (b) TAED- and (c) SP-loaded microparticles formed are highly porous with highly ordered interconnected channels. Higher magnification inset images for figures a–c show unique structural features produced with each type of microparticle. Figures (d) unloaded, (e) TAED, (f) SP show the changes in the TIPS microparticles morphology after they were hydrated for 28 days in minimum essential medium eagle (EMEM media). Higher magnification (inset) show a more open pore structure due to the bulk erosion of the polymer matrix.

TIPS microparticles investigated in the study had a mean diameter of 383 ± 41 μm. Scanning electron microscopy revealed unloaded microparticles prepared from 4 wt% PLGA had highly ordered interconnected pores, with a single larger on the surface of each microparticle (Fig.2a–c). At higher magnification the morphology of the PLGA TIPS particles had a highly anisotropic channel-like structure with an internal ladder-like shape, similar to that previously described [24]. The exterior of the unloaded microparticles contained pores between 1 and 5 μm arranged in a chevron like pattern. Microparticles loaded with TAED exhibited noticeably different surface morphology and pore size. Microparticles loaded with SP appeared to be more porous compared with the unloaded microparticles, possibly due to the small amount of water introduced to the polymer mix to dissolve the SP.

Particle morphology transformed after hydration for 28 days in Eagle’s minimum essential medium (EMEM media) due to PLGA degradation and the release of the TAED and SP compounds from the PLGA matrix due to diffusion and hydrolysis (Fig.2d–f).

**Fig. 3:**
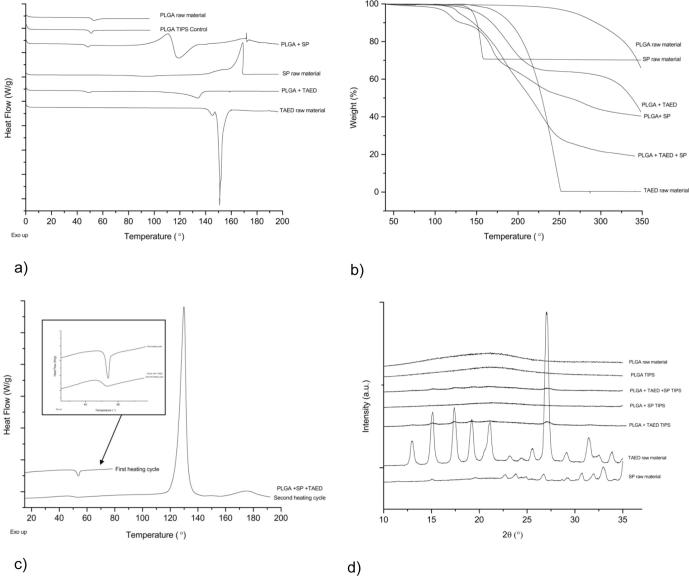
Thermal characterization of TIPS particles. (a) Differential scanning calorimetry – DSC, (b) Thermogravimetry – TGA and (c) reheating SP and TAED loaded TIPS in DSC, (d) Powder X-ray Diffraction – PXRD data for Control, TAED and SP TIPS microparticles and the compounds in their raw format.

PLGA thermograms obtained by differential scanning calorimetry (DSC) showed glass transition temperatures (*T_g_*) of 48.8 °C and 50.2 °C for unloaded TIPS microparticles and pure PLGA polymer, respectively, which is in accordance with reported values in the literature [22], [31]. The characteristic enthalpy of relaxation endotherm overlapped with the *T_g_* (Fig.3a). The slight difference in the *T_g_* could relate to moisture content reflected in a mass loss in the thermogravimetric analysis (TGA) of 2.3% and 1% for unloaded microparticles and pure PLGA polymer, respectively in the temperature range up to 200 °C (Fig.3b). The *T_g_* value of the microparticles loaded with SP was slightly lower than unloaded microparticles despite having a mass loss of 7% up to 200 °C. SP appeared to be stable within the temperature range for studying the PLGA *T_g_* (up to 120 °C) but showed a rapid mass loss indicating degradation with TGA (onset 152 °C, 29% up to 200 °C), with the loss increasing to 36% when incorporated into the PLGA microparticles. The DSC thermogram of SP clearly showed an exotherm indicating the thermal degradation of this compound, and the shoulder showing at the beginning of the exothermic peak indicated its multi-step nature that is characteristic of SP [27]. This exotherm was also observed in the DSC thermogram of PLGA TIPS particles containing SP, which was followed by an endotherm. The presence of PLGA appears to affect the kinetics of the thermal degradation of SP resulting in a decrease of the onset temperature of this chemical reaction as well as showing the endothermic part of the reaction. The DSC signal was the sum of the overlapping endothermic and exothermic processes at a specific temperature range. Therefore, any change to these processes will have resulted in a change to this overall signal.

TAED showed two endotherms with peak values of 145 °C and 150 °C but only one endotherm with a peak value of around 134 °C, indicating melting (Fig.3a). The *T_g_* microparticles loaded with TAED (47 °C) was slightly lower than the unloaded microparticles. This could indicate an interaction between TAED and PLGA polymer resulting in a lower *T_g_* for PLGA and melting temperature (*T_m_*) for TAED. The TGA data depicted in Fig.3b indicate that TAED showed a mass loss of 45% appearing as a single transition with an onset of 187 °C. This changed to a two-step mass loss in the microparticles loaded with TAED, with onset occurring at 155 °C and 322 °C corresponding to a mass loss of 27% and 30%, respectively.

Powder X-ray diffraction (PXRD) patterns (see Fig.3d) revealed unloaded microparticles to be amorphous, similar to the starting raw polymer, whereas SP and TAED in their raw form had crystalline patterns. The microparticles loaded with TAED had clear peaks corresponding to those of TAED, which indicates that the lowering of *T_m_* of TAED loaded particles was indeed caused by interaction between PLGA and TAED and not because of crystallisation of TAED as a different polymorph. However, the microparticles loaded with SP did not show peaks corresponding with the raw SP. DSC reheating of unloaded microparticles just above 50 °C showed the transition to be reversible, confirming glass transition and that PLGA in the microparticles remained amorphous (Fig.3c). The DSC thermogram of TIPS particles containing both TAED and SP also showed an effect of the presence of PLGA and TAED on the exothermic peak of the SP degradation. As explained earlier, the DSC signal was the sum of both exothermic and endothermic process and thus the endothermic peak of the TAED melting is not visible.

### 3.2 Loading efficiency of TAED and SP in TIPS microparticles

**Fig. 4:**
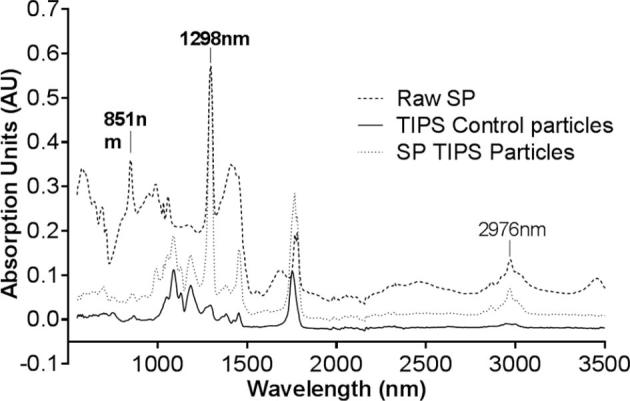
Absorbance spectra of raw SP, unloaded microparticles and SP loaded microparticles. The loading of SP into the microparticles was confirmed by Attenuated Total Reflection Fourier Transform Infrared Spectroscopy (ATR-FTIR) – Perkin Elmer series 2000. The raw SP spectrum has distinct peaks at the wavelengths of 1298 nm and 2976 nm. These peaks were also present at the spectrum of SP loaded microparticles but were not present in the unloaded microparticles.

The theoretical loading of TAED and SP particles was 35.5% (w/w) based on the quantity of precursor compound added to the polymer solution during manufacture. These values were based on the assumption that all of the compound was loaded into the microparticles during manufacture. The experimental loading efficiency measured for TAED was in agreement with the theoretical calculation (35.8% ± 1.15 w/w), whereas the value for SP was 3.95% ± 0.05 w/w. The use of water as a solvent for SP during microparticle manufacture will have resulted in its decomposition to SC and H_2_O_2_. Comparison of the absorbance spectra obtained using Attenuated Total Reflection Fourier Transform Infrared Spectroscopy (ATR-FTIR) for raw SP and microparticles loaded with SP indicate some H_2_O_2_ entrapped may have reverted back to SP as the water and solvent was removed from polymer matrix during lyophilisation (Fig. 4).

### 3.3 Validation of neutraliser efficacy and toxicity

In order to quench the activity of the test antimicrobial agent (i.e. H_2_O_2_ and PAA) after a particular exposure time, a neutralising agent was introduced to the assay at a pre-determined time-point to render the antimicrobial agent inactive. Prior to conducting antimicrobial activity assays, the neutralising agent was validated to assure this was effective in inactivating the H_2_O_2_ and PAA. Furthermore, the neutralising agent may pose toxicity to the target organisms at the concentration used. Therefore a toxicity assay was performed against MRSA and CREC.

Neutraliser efficacy (NE) and neutraliser toxicity (NT) were calculated as: NE = M_A_/M_B_, and NT = M_B_/M_C,_ where M_A_, M_B_ and M_C_ represent the mean number of spores recovered from preparations (A), (B), (C) and control populations (D) respectively. Results were based on 10 replicate test samples. Using documented criteria [26], the neutraliser efficacy and toxicity ratios were calculated as being ≥0.75 for all test-preparations (results not shown) demonstrating that the neutraliser was effective against H_2_O_2_ and PAA and non-toxic to the test organisms.

### 3.4 Antimicrobial activity of TIPS microparticles

**Fig. 5:**
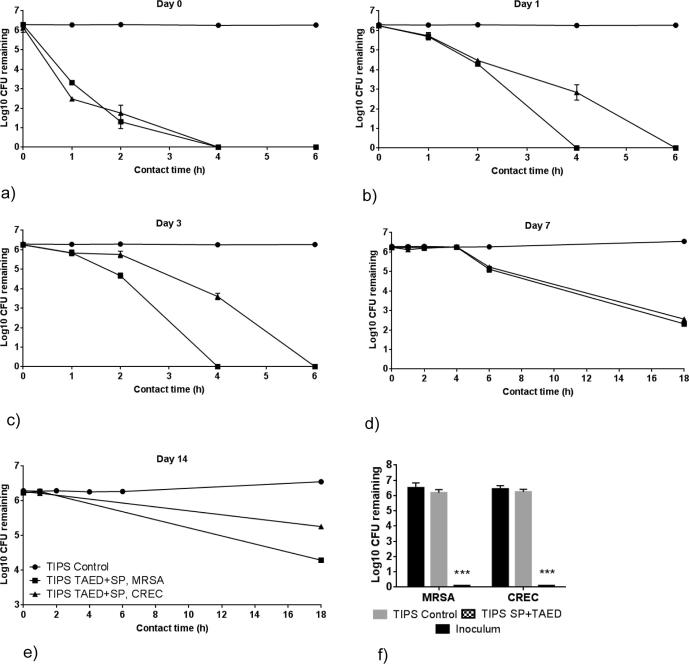
Antimicrobial activity of the microparticles loaded with TAED + SP against CREC and MRSA for 14 days. The antimicrobial activity for the hydration times (a) 1.5 h (p < .05), (b) 1 day (p < .01), (c) 3 days (p < .05) (d) 7 days (p, .05) & (e) 14 days (ns) and different contact times. Hydration time is the amount of time the samples are incubated in complete EMEM medium before supernatant samples are collected. Contact time is the amount of time the supernatant is incubated with bacteria. The TAED + SP TIPS microparticles have antimicrobial activity that lasts for up to 14 days. All data points include error bars. (Some error bars are smaller than data point symbols.) Statistical evaluation was performed by the Friedman test. (f) The loaded microparticles are bactericidal and not bacteriostatic. A bacterial inoculum challenge that had been exposed for 6 h to the active supernatant collected from the loaded microparticles and incubated for 48 h did not show any sign of growth. Two-Way ANOVA with Dunnett's test for multiple comparisons was used to examine if there was a significant difference between the groups. The p values shown refer to test samples compared with untreated inoculum.

A series of studies were performed to evaluate the antimicrobial activity of TIPS microparticles loaded with SP and TAED. Time kill assays measure the time taken for an antimicrobial compound to kill test bacteria. The rate of kill by stabilised PAA was first demonstrated at the minimum bactericidal concentration *(*MBC) values for test microorganisms using an inoculum of 1 × 10^6^ mL^–1^ for each test bacteria. The time kill for MRSA and CREC are shown at Fig. 5.

To simulate *in vivo* application, samples containing an equal quantity of SP and TAED TIPS microparticles were initially hydrated by placing in culture medium to expose the loaded pre-cursor compounds to an aqueous environment resulting in release of the oxidative species. At pre-determined time-points, aliquots of the hydrated microparticles were challenged ∼10^6^ cfu bacteria. Loaded microparticles hydrated for 1.5 h significantly reduced the numbers of CREC and MRSA inoculum challenge (∼6 log10 cfu bacteria) to undetectable levels within 4 h of contact time (Fig.5a). Antimicrobial activity of the loaded microparticles continued when pre-activated for 1, 3 and 7 days, with a statistically significant reduction in the inoculum challenge occurring within 18 h of contact time (Fig.5b–d). At 14 days hydration, the loaded microparticles continued to display a similar trend for antimicrobial activity (∼2 log_10_ cfu and ∼1 log_10_ cfu reductions for MRSA and CREC bacteria, respectively) after 18 h contact time (Fig.5e).

The minimum inhibitory (MIC) and bactericidal concentration (MBC) were measured to determine whether microparticles loaded with TAED and SP were bacteriostatic or bactericidal. The MIC/MBC ratio was found to be 18.33 ± 1.77 ppm and 21.11 ± 2.20 ppm for CREC and MRSA bacteria, respectively. MRSA and CREC titres of ∼6.0 log_10_ cfu were reduced to below the detection limit (10 cfu) within 6 h of contact when exposed to microparticles loaded with TAED and SP. Further confirmation that the loaded microparticles were bactericidal was provided by culturing the washed pellet re-suspended in sterile PBS in agar plates after exposure to the active supernatant for 6 h, which resulted in no detectable bacteria growth (Fig.5f).

### 3.5 *In vitro* and *in vivo* biocompatibility

**Fig. 6:**
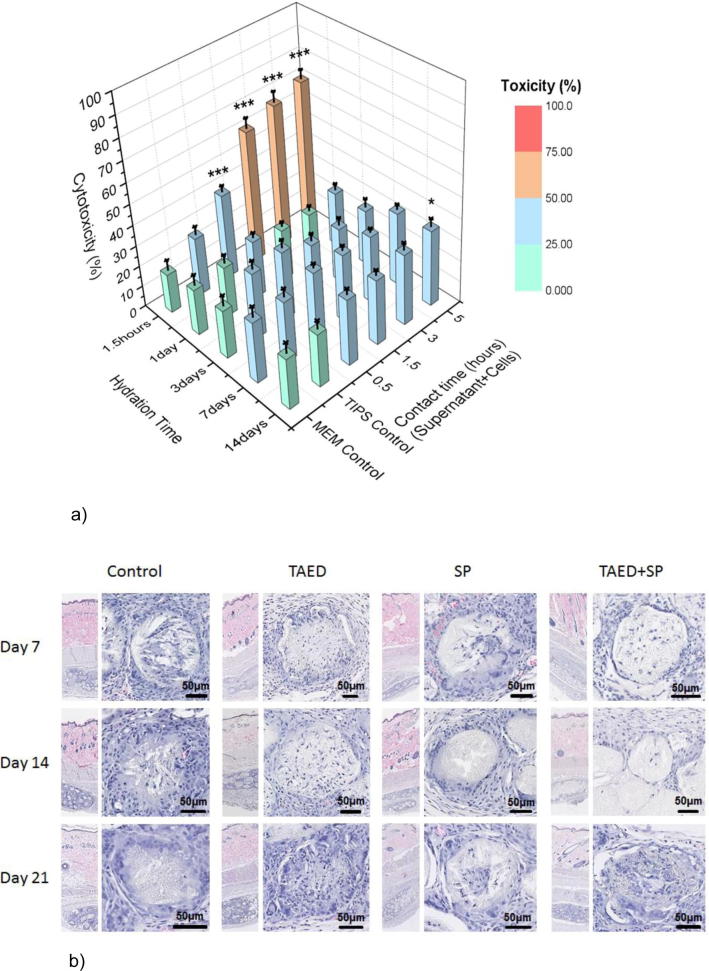
Biocompatibility of TAED and SP when incubated with cells or injected into rats. (a) Cytotoxicity of supernatant collected from loaded and unloaded microparticles after different times of hydration and contact. No significant increase in cytotoxicity to L929 cells was measured for the majority of incubation conditions. An increase occurred at 1.5 h hydration time and for contact times from 0.5 to 5 h if compared to EMEM Control (p < .001). A Two-Way ANOVA with Dunnett's test for multiple comparisons was used to examine if there was a significant difference between the groups. The p values shown refer to test samples compared with untreated cells incubated in medium only. (b) Histology of the implant site after 7, 14 and 21 days. No discernible difference in the tissue response was observed between the unloaded microparticles and those loaded with TAED and SP. The microparticles lose their structure over time and become infiltrated by inflammatory cells and fibrovascular tissue that forms part of the expected tissue response continuum as the microparticles degrade.

Preliminary toxicological assessment of the oxidative species released from the loaded microparticles was performed *in vitro* using a mouse fibroblast cell line (L929). The purpose of the cytotoxicity test was to determine the biological reactivity of mammalian cell lines following contact with the oxidative species released from the loaded microparticles over a range of time points. Supernatant collected from the hydrated microparticles at pre-determined intervals was incubated with the fibroblasts. For the majority of incubation times, the level of cytotoxicity (based on measuring changes in membrane integrity that occur as a result of cell death) was not elevated above background levels obtained for cells alone or cells incubated with supernatant samples collected from unloaded microparticles. Increased cytotoxicity occurred only with supernatant samples collected before 1.5 h hydration of the loaded microparticles and incubation of the supernatant with cells for 1.5–5 h (Fig.6a).

The biocompatibility of TIPS microparticles loaded with SP and TAED was investigated *in vivo* using an intracutaneous reactivity test to determine the potential for causing localised reaction and irritation. Subcutaneous injection of the microparticles into rats was well-tolerated with no clinical or local sign of erythema and edema at the implant site at any of the time-points. Histology of the implant site revealed the microparticles to be present at all experimental time points, eliciting a minimal or slight granulomatous inflammatory reaction (Fig.6b).

## 4 Discussion

The porous structure observed in unloaded TIPS microparticles was created by dimethyl carbonate (DMC) solvent crystals formed when the droplet of polymer solution froze as it entered the liquid nitrogen quenching bath. The frozen solvent was removed by lyophilisation resulting in the highly porous polymer structure. The different surface morphology observed with microparticles loaded with TAED was due to the introduction of acetonitrile (ACN) to the polymer solution as a solvent for TAED. The melting temperature of ACN (−45 °C) and the interaction with the polymer solution is likely to have changed the formation of solvent crystal shapes in the frozen droplet structure [28]. The increased porosity observed with microparticles loaded with SP is likely to have resulted from the inclusion of water introduced to the polymer mix to dissolve the SP. This may have caused changes to the thermal energy of the system, altering the shape of the solvent crystals formed in the droplet during the freezing process [28]. Mixing aqueous solutions into a hydrocarbon-rich polymer is challenging [29]. It is likely only limited quantities of H_2_O_2_ produced from SP dissolved in water during manufacture would become entrapped in microparticles due to its low molecular weight, the hydrophobicity of the polymer, and the expected high diffusion coefficient from the polymers matrices [29]. Further decomposition of H_2_O_2_ into water and oxygen whilst in the aqueous solvent and polymer solvent (DMC) is likely to have occurred [29], [30]. As SP is an adduct with hydrogen peroxide it would be expected to reform during the lyophilization process if the hydrogen peroxide has not degraded. Some of the H_2_O_2_ formed may also have been removed along with water from the microparticles during lyophilisation, similar to that observed in previous studies [31]. However, the microbiology data indicate the quantity of SP loaded is sufficient to achieve biocidal activity. Further studies are necessary to characterize the behaviour of SP during the manufacturing process.

The antimicrobial activity of TAED and SP is attributed to the formation of PAA together with its anions via the reaction of the acyl group in TAED with the hydroperoxide anion from SP. PAA has a higher oxidation potential and stronger nucleophilic abilities than H_2_O_2_ produced by SP alone [20], [32]. H_2_O_2_ is active against a wide range of microorganisms, including bacteria, yeasts, fungi, viruses and PAA inactivates Gram-positive and Gram-negative bacteria, bacterial spores, fungi, and yeasts at lower concentrations than H_2_O_2_ [20], [21], [33]. The current feasibility study used MRSA and CREC as representative Gram positive and Gram negative models, respectively, to gauge the efficacy of oxidative biocide-precursor compounds released from TIPS microparticles. Further studies that reflect polymicrobial environments will be necessary to determine whether the current technology is suitable as a broad-spectrum antimicrobial approach. It should be noted that, to date, there is no evidence showing bacterial resistance or tolerance to oxidative biocides. This can be attributed to the mechanism of action of oxidative biocides that involves degradation of many components of the cell, including membrane lipids, proteins and genomic material, thus reducing the likelihood of bacterial resistance.

For clinical acceptance, the oxidative species must be efficacious whilst not eliciting to tissue damage. Existing modes for delivering oxidative species in clinic, such as high concentrations of H_2_O_2_ (3%/∼1.2 M) as a topical antiseptic for surgical wounds, notably in endodontics and periodontitis [34], risk causing collateral tissue damage and are unsuitable for treating infected wounds with a large surface area or where repeated application is necessary [7]. The *in vitro* cytotoxicity observed was lower than expected since PAA and H_2_O_2_, formed by the microparticles loaded with TAED and SP, are strong oxidising agents that can cause tissue damage at high concentrations. Previous studies investigating the cytotoxicity of different concentrations of H_2_O_2_ have indicated the cytotoxic effects on cells are both dose-dependent and time-dependent [8], [35]. We observed a similar trend, with increased cytotoxicity occurring in samples collected from microparticles after shorter periods of hydration combined with longer contact time between the supernatant and cells. This time point was primarily included in the current proof of concept study to identify the boundary conditions within which effective and safe use of the microparticles is likely to be achieved. However, it should be noted that the incubation conditions used for this time point are not truly reflective of exposure to the oxidative biocides likely to occur *in vivo*. Unlike *in vitro* conditions, implantation *in vivo* will expose the microparticles to a dynamic physiological environment where tissue perfusion is likely to prevent a build-up of the biocidal compounds that occur *in vitro* under static conditions over 5 h. The process used to fabricate the microparticles is amendable to further optimization in terms of polymer degradation and release of the precursor compounds, as well as the amount of compounds loaded into microparticles. This provides scope for future studies to adjust the release of oxidative biocides to match requirements associated with different uses.

*In vivo* implantation revealed evidence of the expected tissue-response continuum to microparticle degradation at all time-points [22], [23]. No discernible difference in the response was observed between groups treated with a combination of the loaded microparticles and those containing the individual precursor compounds or unloaded control microparticles. Although the earliest histology time point was beyond the time point for peak release of oxidative biocide *in vitro*, the lack of any signs of an adverse tissue reaction, such as necrosis, that would be expected in response to toxicity caused by oxidative biocides suggests the precursor-loaded microparticles were well tolerated. The data collected to date concur with the low toxicity effects observed *in vitro* and are supported by existing data on the low risk of acute toxicity associated with SP and TAED [36], [37]. Future studies need before clinical translation will investigate whether the quantities of precursor compounds loaded into the microparticles are efficacious in tackling bacterial infections using pre-clinical *in vivo* models that reflect polymicrobial environments, such as chronic wounds related to diabetic foot ulceration or orthopaedic implant surgery. Likewise, further pre-clinical testing will be aimed at verifying non-toxicity of SP and TAED associated with long-term exposure to SP and TAED that occurs whilst the microparticles completely degrade, as well as their antimicrobial activity beyond 14 days demonstrated in the current study.

The TIPS microparticle technology provides an effective delivery vehicle for targeted and controlled release of the peroxygen donor, SP, combined with an acetyl donor, TAED, resulting in delivery of antimicrobial activity via a dynamic equilibrium mixture of H_2_O_2_ and PAA. The hydrophobic polymer PLGA shelters the precursor compounds from the aqueous tissue environment found *in vivo*. Degradation of the PLGA via hydrolysis in aqueous environments provides sustained delivery of non-toxic concentrations, which counters rapid clearance and decomposition of the oxidative species, thus avoiding the need for repeated treatment to maintain an effective local concentration.

## 5 Conclusions

Antibiotic resistance poses a significant risk to human health; therefore, novel approaches to combat infection are urgently needed. Re-evaluation of existing small molecules with well-established biocidal properties and a low propensity to give rise to resistance, especially in terms of optimising their mode of delivery, could offer an accelerated pathway to clinical application for a range of new therapeutic products.

The current study demonstrates for the first time it is possible to deliver PAA and H_2_O_2_ with localised potent, non-toxic antimicrobial activity using pre-cursor compounds TAED and SP loaded into TIPS microparticles. The TIPS technology can provide an innovative antimicrobial alternative to classic antibiotic agents and could serve as adjunctive or replacement therapy. Although our study reports the use for topical treatments, it is reasonable to expect that the technology could be adapted for oral or systemic delivery. The availability of a wide range of acetyl donors and a smaller range of peroxygen donors, combined with significant design variation for the particles themselves (including rate of degradation via hydrolysis), offers great potential for the creation of bespoke approaches to different clinical conditions.
